# The first case report of dental floss pick-related injury presenting with massive hemoptysis: A case report

**DOI:** 10.1186/1752-1947-2-78

**Published:** 2008-03-11

**Authors:** Chun-Ta Huang, Chao-Chi Ho, Yi-Ju Tsai, Pan-Chyr Yang

**Affiliations:** 1 Division of Pulmonary Medicine, Department of Internal Medicine, National Taiwan University Hospital, Taipei, Taiwan, No. 7, Chung-Shan South Rd, Taipei 100, Taiwan; 2 Department of Emergency Medicine, National Taiwan University Hospital, Taipei, Taiwan; 3 School of Medicine, College of Medicine, Fu Jen Catholic University, Taipei, Taiwan, 510 Chung Cheng Rd , Hsinchuang , Taipei County 24205, Taiwan

## Abstract

**Introduction:**

A tracheobronchial foreign body is a rarely mentioned cause of massive hemoptysis. Although an aspirated toothpick is a well-known cause of traumatic injury to the respiratory tract, a similar device called a dental floss pick, which is much larger than a toothpick, has never been described as a tracheobronchial foreign body.

**Case presentation:**

We report a case of massive hemoptysis in a 32-year-old man due to a dental floss pick in the left main bronchus. Flexible fiberoptic bronchoscopy was successful in removing the foreign body.

**Conclusion:**

Tracheobronchial foreign body can be a medical emergency requiring immediate intervention and massive hemoptysis may be the presenting symptom. Flexible fiberoptic bronchoscopy is recommended as the first-line treatment modality for tracheobronchial foreign body removal. A dental floss pick may present as a tracheobronchial foreign body and can reside in the airway asymptomatically for many years.

## Introduction

Massive hemoptysis comprises only 5 percent of hemoptysis events; however, the mortality rate for patients with massive hemoptysis can be as high as 80 percent. Three major etiologies account for 90 percent of cases: bronchiectasis; tuberculosis; and bronchogenic carcinoma [1,2]; a tracheobronchial foreign body is a rare clinical entity leading to massive hemoptysis [3].

Accidental toothpick ingestion has often been reported as the cause of gastrointestinal and respiratory tract injuries [4,5], and under very rare circumstances, may result in constrictive pericarditis, coronary artery perforation, obstruction of the ureter, and subphrenic abscess [6-9]. Dental floss picks, which are also a plaque remover, are much larger objects than toothpicks, and have never been described as a causative agent of aerodigestive tract injuries. We report herein an adult patient with a dental floss pick stuck in the left mainstem bronchus asymptomatically for 8 years, who presented to the emergency department with acute onset of massive hemoptysis. The dental floss pick was successfully removed under flexible fiberoptic bronchoscopy and soon thereafter the hemoptysis resolved. To our knowledge, this is the first case report concerning a dental floss pick as a tracheobronchial foreign body leading to massive hemoptysis.

## Case presentation

A 32-year-old man with no pertinent medical history presented to the emergency department with acute coughing up of 300 ml of bright-red blood over 3 hours following a sneezing episode. The patient was a taxi driver and had no history of cigarette smoking, alcohol drinking, upper airway complaints, chest trauma, or use of aspirin or non-steroidal anti-inflammatory drugs. Also, he denied prior hemoptysis or other pulmonary symptoms, infectious symptoms, or a family history of hemoptysis or brain aneurysms.

**Figure 1:**
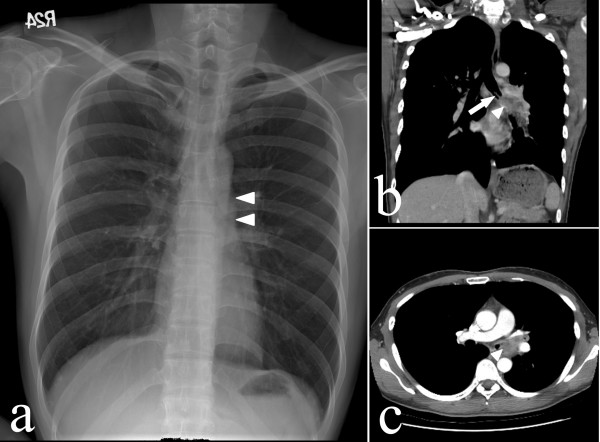
**Posterior-anterior chest radiograph (Panel a) shows an ill-defined opacity around the left hilum (arrowheads).** Chest computed tomography (Panel b and c) shows soft tissue density (arrowheads) within the left mainstem bronchus with a needle-shaped object (arrow) protruding from it.

**Figure 2:**
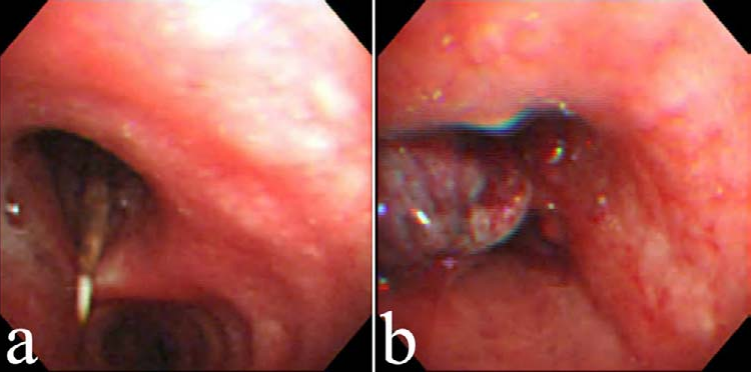
Flexible fiberoptic bronchoscopy revealed a dental floss pick in the left main bronchus (Panel a) and granulation tissue formation after removal of the object with biopsy forceps (Panel b).

**Figure 3:**
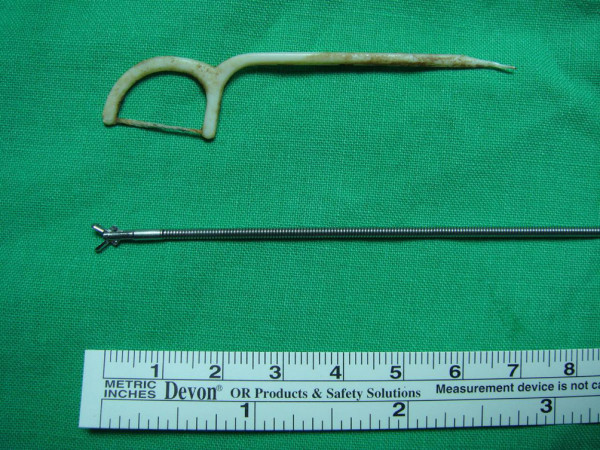
The photograph shows the 7-cm dental floss pick that was successfully removed by flexible fiberoptic bronchoscopy with the biopsy forceps.

His temperature was 36.8 degrees Celsius, pulse was 88 per minute, respirations were 18 per minute, and blood pressure was 128/88 mmHg. Pulse oximetry showed an oxygen saturation of 98% in the room air. The results of physical examination were unremarkable. The complete blood count, the levels of urea nitrogen and creatinine, liver biochemistry, and coagulation profiles were also normal. Urinalysis revealed no abnormalities. A chest X-ray (Figure 1a) showed an ill-defined opacity around the left hilum and chest CT (Figures 1b and 1c) demonstrated soft-tissue opacity within the left mainstem bronchus with a needle-shaped material protruding from it. A retained tracheobronchial foreign body was suspected. Flexible fiberoptic bronchoscopy found impaction of a dental floss pick in the left main bronchus (Figure 2a) with granulation tissue formation (Figure 2b) and clotted blood over it. The object (Figure 3) was successfully removed using biopsy forceps (Figure 3) and no procedure-related complications, such as tracheal laceration, vocal cord injury or bleeding, were noted. After foreign body retrieval, the patient recalled having had dental floss pick ingestion 8 years earlier. He still uses dental floss picks to clean his teeth every day. He no longer had hemoptysis during his hospital stay and he was discharged a few hours later.

## Discussion

Though bronchiectasis, tuberculosis and bronchogenic carcinoma are the three most common causes of massive hemoptysis, a wide variety of disorders or situations could also result in such an event [1-3]. The mechanisms of massive hemoptysis in these disease entities may be different. For example, in pulmonary tuberculosis, rupture of Rasmussen's aneurysm or erosion of a broncholith through a vessel may be the candidate mechanism [10]. In bronchiectasis, chronic airway inflammation leads to hypertrophy and expansion of the peribronchial vessels and rupture of these vessels causes hemorrhage [11]. However, to date, only a few case reports regarding a foreign body as the cause of massive hemoptysis have been presented and the mechanisms by which the foreign bodies cause massive hemoptysis are not well delineated [12-16]. In this patient, we considered that chronic inflammation induced by the foreign body caused hypertrophy of the surrounding vessels and massive hemoptysis developed upon rupture of the vessels.

This case demonstrates a rare complication and the clinical course of a tracheobronchial foreign body. Why did this patient become asymptomatic such a long time after foreign body aspiration and develop massive hemoptysis? The patient was a young and strong man, and had no underlying cardiopulmonary disorders; therefore he had no functional impairment in regular daily activities and no symptoms ascribable to the tracheobronchial foreign body though the granulation tissue around the foreign material partially occluded the lumen of the left mainstem bronchus. Because no other abnormalities were identified on chest CT and the patient had no bleeding tendency or other systemic illnesses, the cause of massive hemoptysis could only be ascribed to the tracheobronchial foreign body. We speculated that the power generated from the sneezing possibly dislodged the foreign body and incurred injury to the adjacent hypertrophied vessels. Consequently, an episode of acute and massive hemoptysis ensued.

Dental floss picks, as its name implies, combine the functions of a toothpick and dental floss, and are widely used to maintain good oral hygiene. Nevertheless, unlike toothpicks or dental floss, they have never been presented as a tracheobronchial foreign body or caused gastrointestinal damage probably because the size is much larger than the other devices. This case demonstrates that large objects like a dental floss pick may be the cause of a tracheobronchial foreign body and reminds everybody to use them cautiously.

Tracheobronchial foreign bodies can be a life-threatening emergency requiring prompt removal; however, they may remain undetected for years causing trivial or nonspecific symptoms. An accurate history and a high index of suspicion are the determining factors leading to a diagnosis of tracheobronchial foreign bodies, but both patients and physicians often neglect the importance of detailing a remote history of foreign body inhalation. Our patient didn't mention this episode until the dental floss pick was removed by flexible fiberoptic bronchoscopy. Therefore, a tracheobronchial foreign body was not considered in the differential diagnosis until chest X-ray or chest CT findings suggested the presence of bronchial foreign body; this was also unusual in that in most instances the foreign material is not discernible by radiographic studies.

Hemoptysis is not an uncommon complication of tracheobronchial foreign bodies and it was observed in between 15 and 23% of patients based on two large case series [17,18]. Massive hemoptysis, defined as the expectoration of more than 100 to 600 ml of blood in 24 hours, may also develop in this situation and is a medical emergency that places the patient at high risk of asphyxia and death [19]. Fortunately, the patient described here didn't suffer from respiratory compromise and the massive hemoptysis stopped spontaneously soon after his arrival at the emergency department. In evaluating such patients, pulmonary infection is the leading cause of hemoptysis worldwide [19]; however, tracheobronchial foreign bodies should also be taken into consideration in certain circumstances.

Hemoptysis is a well-known indication for flexible fiberoptic bronchoscopy [20]; however, not everyone presenting with hemoptysis needs such a procedure. Chronic or recurrent streaky hemoptysis in a patient with chronic bronchitis or bronchiectasis is not a routine indication for bronchoscopy. Diagnostic bronchoscopy should be considered in patients with significant or new hemoptysis; nevertheless, indications for flexible fiberoptic bronchoscopy when a patient presents with hemoptysis and a normal or non-localizing chest roentgenograph continue to be controversial [21].

Flexible fiberoptic bronchoscopy is recommended as the first-line treatment modality for tracheobronchial foreign body removal in the adult population with success rates of more than 90% when performed by an experienced bronchoscopist [5,18]. Compared to rigid bronchoscopy, flexible fiberoptic bronchoscopy has greater visibility and range, can be done outside the operating room, and requires no general anesthesia. However rigid bronchoscopy affords superior airway control, allows for a larger field of view than flexible fiberoptic bronchoscopy, and has the ability to ventilate the patient during the procedure. Given the potential to cause obstruction or injury of the airways upon removal of bulky or sharp tracheobronchial foreign bodies, attempts at removing such objects without backup rigid bronchoscopy are not recommended. Therefore, flexible fiberoptic bronchoscopy should be used in concert with rigid bronchoscopy to provide the most appropriate treatment for the patients with tracheobronchial foreign bodies.

In conclusion, this unusual case emphasizes that dental floss picks may present as a tracheobronchial foreign body and can reside in the airway asymptomatically for many years. Tracheobronchial foreign bodies can be a medical emergency requiring immediate intervention and massive hemoptysis maybe the presenting symptom. For the management of patients with a tracheobronchial foreign body, flexible fiberoptic bronchoscopy should be promptly performed to identify the location of the foreign body and any associated injuries, and in most cases the foreign body can be removed successfully at the same time.

## Conclusion

- Tracheobronchial foreign body can be a medical emergency requiring immediate intervention and massive hemoptysis may be the presenting symptom.

- Flexible fiberoptic bronchoscopy is recommended as the first-line treatment modality for tracheobronchial foreign body removal with success rates of more than 90%.

- A dental floss pick may present as a tracheobronchial foreign body and can reside in the airway asymptomatically for many years.

## Competing interests

The author(s) declare that they have no competing interests.

## Authors' contributions

CTH summarized the case and drafted the manuscript. CCH and PCY participated in the design and coordination of the manuscript. YJT helped to draft the manuscript. All authors read and approved the final manuscript.

## Consent

Written informed consent was obtained from the patient for publication of this case report and the accompanying images.
